# Hospitals in India

**Published:** 1896-07-11

**Authors:** 


### HOSPITALS IN INDIA.

### CIVIL HOSPITALS AND DISPENSARIES IN THE MADRAS PRESIDENCY.

Under the system of medical relief instituted in India by the British Government, treatment of the sick on European lines and belief in European remedies are certainly steadily progressing in favour with the native population. Every year sees the establishment of more dispensaries, and a considerable increase in the number of patients who apply to these institutions for help in time of sickness.

The dispensary system peculiar to India arose in the first place through the necessity of establishing additional hospitals to which civil officers and their staff, the police, and the public could be admitted, and the consequent demands from various parts of the country for branch dispensaries. The Government then provided funds for the erection of new institutions where needed, the expenses when established being p ovided for by local municipal funds, subscriptions, patients' payments, and from other miscellaneous sources. From the beginning it was the object of the Government of India to put all such institutions for the care of the sick under local control, subject to proper inspection (submission to Government inspection being made a condition of obtaining grants from public funds), and by facilitating native medical education to finally break down prejudices and accomplish what has been described by a native of India as the "great and unique experiment of engrafting a Western civilisation upon an Eastern stock." To this end free and assisted training is given at certain medical schools, and scholarships and prizes awarded to encourage the men and women of India to enter the medical profession.

Dispensaries, it must be understood, are not in India merely institutions for giving outdoor relief; they are also practically hospitals, many if not most of them containing a certain number of beds. Official inspections are fairly frequent, and the latest report from the Madras Presidency contains a new feature in the full text of the comments of the Surgeon-General on the existing condition of the chief hospitals and dispensaries visited by him. The tale therein told of unsuitable buildings, lack of sufficient accommodation and nursing, and instruments "actually dirty," appearing "not to have been cleaned for years," seems to show need for much internal reformation.

The proportion of hospitals and dispensaries to the population in Madras is higher than in the Punjab or the North-West Provinces, being in the former 1 to 72,592, while in the North-West Provinces the figures stand at 1 to 142,698, and in the Punjab 1 to 85,495. The number of institutions included in the annual reports have increased largely of late years, and for 1894 shows a total increase for the year of 20. Over 93,000 more women were treated in that year than in the one preceding, a fact which is in itself very satisfactory.

How best to help the women in India, morally and physically, has been a question in which much interest has been taken throughout by their sisters in England. From the Queen's personal request to Lady Dufferin to inquire into the matter has sprung the Lady Dufferin Fund, which has the double object of providing medical relief and encouraging medical education. Native ladies are badly wanted to carry on the work, but the ranks of these women students are but slowly recruited, for the difficulty of overcoming caste prejudices is one which can be only accomplished by time and patience. Medical women are everywhere in demand, as is evidenced by the Madras report, where, in several instances, it is stated that proposals have been made to employ "lady apothecaries," but none are available. In every case where lady medical practitioners are in charge of separate institutions satisfactory progress is reported, and the native women are becoming encouraged to take advantage of the help offered to them.

More trained midwives are in urgent request in some of the Madras districts, the medical officers of the Government Lying-in Hospital, and Sir S. Ramasawmy's Lying-in Hospital, Madras, having been applied to in vain for qualified women. Yet the report of the work accomplished during the year is certainly encouraging, and points to a steady increase in the number of women trained for this all-important branch of the work and in the cases attended and succoured.

With regard to the general statistics for the year, perhaps the subjoined table will put the figures most clearly before the reader. The death-rate throughout the Presidency has fallen satisfactorily since 1892, though deaths from "septic causes acquired in hospital" are too frequent, and the reasons are not far to seek, as a study of the criticisms on the condition of some of the institutions referred to above will show. Special attention is drawn in the report to the number of successful operations performed, the death-rate being 2·7, whilst at the same time it is suggested that the surgical side of medical practice is not developed as it should be in some districts. Skilful surgery undoubtedly does much to make hospitals popular, and encourages the people to apply to them for aid.

The total expenditure on civil hospitals and dispensaries in the Madras Presidency during 1894 amounted to a sum total of Rs.10,07,983.15.9. Of this amount Government contributed Rs.98,160.5.5, the rest coming from municipal and local funds, subscriptions, paying patients, and other miscellaneous sources. Private subscriptions have fallen off during the last twenty-five years, in 1869 amounting to Rs.49,750.10.1, while the average for the last four years, 1890 to 1894, has been Rs.20,433.11.4. The sums received in payment from patients are comparatively small, smaller than might reasonably be expected, though it must be a difficult matter to impress upon the native mind any due sense of their obligations on this point.

The chief facts to be gleaned from a study of the progress of medical labours in India of late years are, in the first place, that native prejudice is being steadily, if slowly, overcome; and secondly, that the great need now is for more material in the shape of men and women, to carry on the work begun and spread the benefits of their knowledge and training far and wide.

| Years. | Number of Patients treated. | | Daily Average Attendance. | | Number of Beds available. | Mortality among In-patients (per centage). | Number of Operations. | | Total Expenditure. |
|---|---|---|---|---|---|---|---|---|---|
| | In. | Out. | | | | | Major | Minor | |
| | | | | | | | | | Rs. |
| 1892 | 39,741 | 3,066,645 | 1,783·9 | 19,487·[illegible] | 2,985 | 9.35 | 4 391 | 108,149 | 8,91,183 |
| 1893 | 39,579 | 3,291,391 | 1,676·7 | 20,669·8 | 3,088 | 7·69 | 5,567 | 118 314 | 8,89,420 |
| 1894 | 41,954 | 3,693,315 | 1,742·7 | 22,496·5 | 3,236 | 6·62 | 5,874 | 129,469 | 10,07,984 |

